# Assessing the impact of mental health capacity building of Primary Health Care (PHC) personnel

**DOI:** 10.1192/j.eurpsy.2022.1589

**Published:** 2022-09-01

**Authors:** N. Makhashvili, T. Latibashvili, R. Badriashvili, L. Gaprindashvili

**Affiliations:** 1 Ilia State University, Mental Health Resource Centre, Tbilisi, Georgia; 2 GIP-Tbilisi, Club Synergy, Tbilisi, Georgia

**Keywords:** Mental health Burden, Primary Healthcare, Capacity Building

## Abstract

**Introduction:**

COVID-19 poses an immense challenge to health systems and societies, associated with a burden of mental health in the population. The pandemic is uncovering treatment gaps in mental health systems, especially in Low and Middle-Resource Countries, as Georgia. The high burden calls for renewed efforts to integrate mental health into Primary Health Care (PHC) to address increased mental health needs of the population. The capacity building of PHC personnel is ongoing since October 2020, according to mhGAP algorithm. Family doctors (FD) are trained in identification and management in priority mental conditions.

**Objectives:**

The overall aim of the study was to assess the impact of capacity building of PHC personnel. This was an implementation research seeking to understand how effective was the offered capacity building process and what could be lessons learnt.

**Methods:**

We employed a mixed-methods process evaluation design utilising a series of instruments specifically designed to provide data for the domains as training/capacity building, service delivery and user satisfaction.

**Results:**

FD were able to identify the most prevalent conditions - Anxiety (74%) and Depression (39%); in 22.8% the comorbidity was recognized. The psychoeducation was the most common method of management used by 72%. In 39.4% FD were able to recommend at least one medicine to their patients. 83.3% of patients reported improved conditions.

**Conclusions:**

The family doctors are able to identify and manage certain mental health conditions after proper trainings and regular supervision. This study has simultaneously identified targets for change within the broader mental health system.

**Disclosure:**

No significant relationships.

